# Investigating the fire dynamics of mounted PV weathering effects and material changes

**DOI:** 10.1016/j.isci.2025.113410

**Published:** 2025-08-29

**Authors:** Muhsin Mohamed Baseer Ahamed Mohamed, Ye Xian Ang, Rhonda Jia Hui Tan, Li Song Tung, Xingchi Xiao, Maloy Das, Leonard Wei Tat Ng

**Affiliations:** 1 School of Materials Science and Engineering, Nanyang Technological University, Singapore, Singapore

**Keywords:** Energy engineering, Materials science

## Abstract

Solar photovoltaic (PV) systems constitute approximately 37% of global renewable energy capacity, yet their fire safety under environmental degradation remains inadequately understood. PV backsheets serve as the primary interface between external fire sources and modules, making their long-term fire performance critical for system safety. This study systematically quantified flame spread behavior on weathered PV backsheets. Two commercial backsheet types underwent accelerated weathering for up to six weeks, followed by comprehensive characterization using Fourier transform infrared (FTIR) spectroscopy, differential scanning calorimetry (DSC), thermogravimetric analysis (TGA), scanning electron microscopy (SEM), and tensile testing. Fire performance was assessed using a time-to-marker (TTM) methodology to measure flame propagation rates. Six-week weathered samples exhibited a 46% faster flame spread, demonstrating significant degradation in fire resistance. Chemical analysis revealed polymer chain scission and formation of degradation products, while mechanical testing showed up to 18% reduction in tensile strength. These findings highlight critical gaps in current safety standards and demonstrate the importance of incorporating weathering effects into PV fire safety assessments for long-term system reliability.

## Highlights

• Weathered PV backsheets show 46% faster flame spread after 6 weeks
• Chemical degradation correlates with reduced fire resistance
• TTM methodology quantifies weathering effects on flame propagation
• Current safety standards inadequately assess aged PV components

## Graphical abstract

**Figure:**
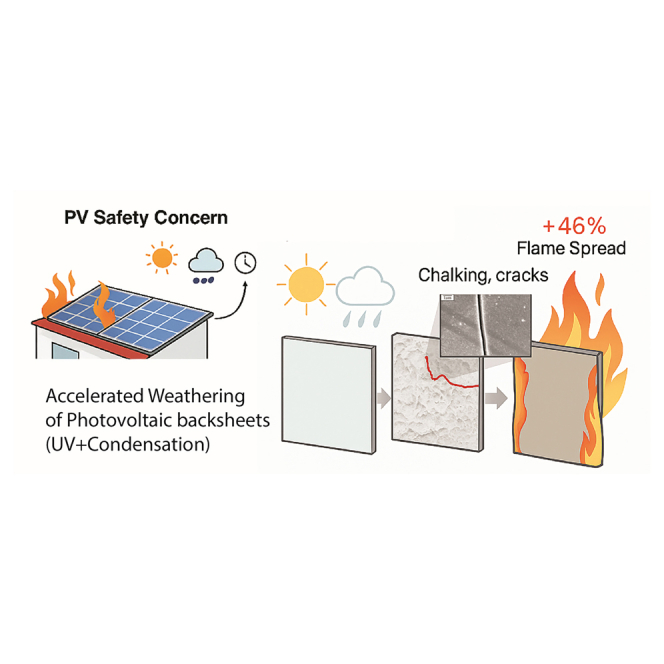

## Introduction

**Figure 1:**
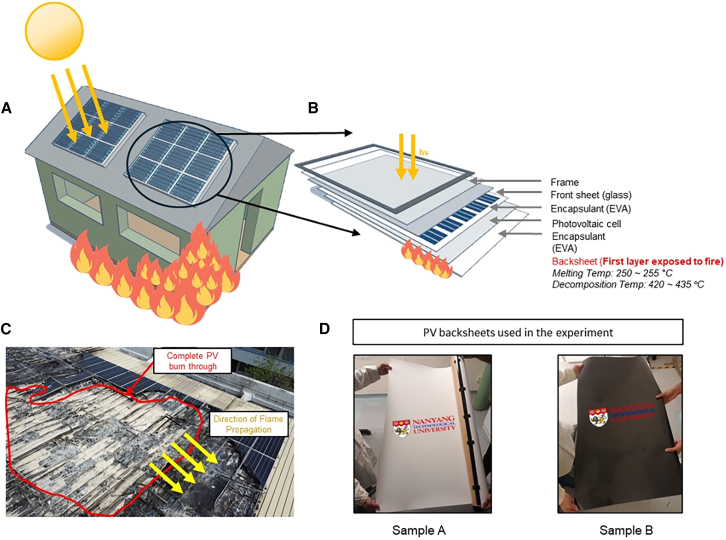
PV module fire dynamics (A) Schematic of building-to-PV module fire spread. (B) Cross-section of standard PV module showing backsheet position. (C) PV fire incident in Singapore showing char patterns indicating low-temperature combustion regions. (D) PET-based PV backsheet samples used in this study.

Photovoltaic (PV) systems have emerged as a cornerstone of global renewable energy strategies, driving the transition toward sustainable power generation. However, the rapid increase in deployment of these systems has brought to light critical safety concerns, particularly regarding fire risks. From 2017 to 2018, PV fire-related incidents saw a striking increase,^1^ necessitating improved understanding of fire dynamics in these installations. Recent comprehensive studies have further documented various types and causes of PV-related fires across different regions,^2^ underscoring the urgent need for enhanced safety measures and understanding of fire dynamics in PV installations. PV-related fires present unique challenges for suppression efforts due to modified fire dynamics on roof structures^3^ and can spread extensively through adjacent modules (Figures 1C and S1–S3).

Fire propagation in PV systems is influenced by multiple factors, including installation geometry and component combustibility.^4^ The gap height between modules and rooftop surfaces significantly affects fire spread rates,^5^ while the fire performance of individual components is evaluated using standardized flame spread ratings such as American Society for Testing and Materials (ASTM) E84. In a building-to-PV module fire scenario (Figure 1A), the backsheet layer typically ignites first, acting as a critical fuel source for propagation.

PV backsheets generally comprise a polyethylene terephthalate core with various configurations on air and cell sides, including Tedlar/Polyester/Tedlar (TPT), Kynar/PET/Kynar (KPK) and Tedlar/PET/EVA (TPE).^6^ These configurations offer different combinations of durability and protection properties. Despite their critical role as the initial flame-exposed layer (Figure 1B), backsheet fire performance remains insufficiently characterized, particularly regarding degradation from environmental exposure. Current safety standards predominantly evaluate entire modules rather than specific components, potentially overlooking vulnerability points.

The limited research on PV backsheet fire properties includes work by Nair and Kulkarni,^7^ though their study does not address weathering effects. Our investigation specifically targets this knowledge gap by examining weathered backsheets. While several studies have examined weathering processes in PV materials,^8^ their specific impact on fire performance remains understudied. We selected backsheets for this study based on their position as the primary interface between external fire sources and the PV module.

This research quantifies fire performance changes in weathered PV backsheets through a systematic laboratory investigation. Two types of commercial backsheets were subjected to accelerated weathering for periods up to six weeks, characterized through multiple analytical methods, and evaluated for flame spread behavior. Our methodology introduces an approach using the time-to-marker (TTM) concept to precisely measure flame propagation rates across weathered samples. The study objectives were to (1) analyze compositional changes resulting from environmental exposure and (2) establish correlations between these changes and flame spread characteristics. The findings demonstrate significant alterations in fire performance post-weathering, with implications for PV safety protocols and standards.

## Results

**Figure 2:**
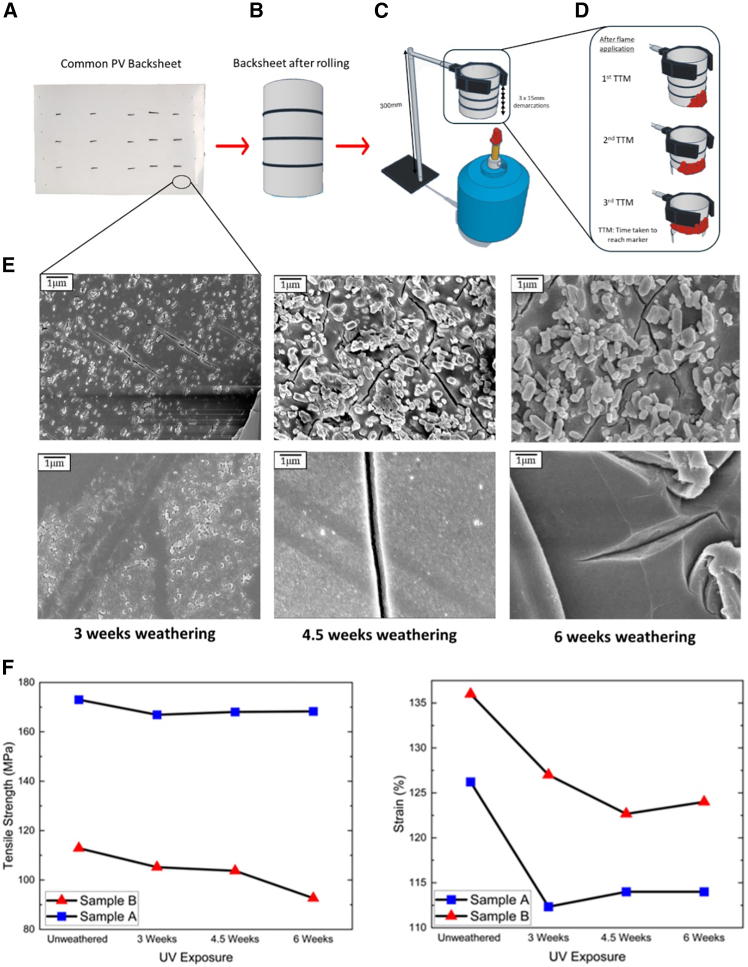
Experimental procedures and material characterization (A) Sample preparation after weathering. (B) Flame test sample preparation. (C) Experimental setup. (D) Time-to-marker measurement points. (E) SEM images showing chalking and cracking effects on weathered backsheets. (F) Tensile test results for samples A (TPE PV backsheet) and B (KPK PV backsheet) after weathering. The non-linear strain patterns observed, particularly the increase at 4.5 weeks, reflect competing cross-linking and chain scission mechanisms.

Two commercial PV backsheet types (TPE and KPK configurations) were subjected to accelerated weathering using UV radiation and condensation cycles for up to six weeks (Figure 2A). Samples were characterized using multiple analytical techniques including Fourier transform infrared (FTIR) spectroscopy, differential scanning calorimetry (DSC), thermogravimetric analysis (TGA), scanning electron microscopy (SEM), and tensile testing to assess chemical, thermal, and mechanical property changes (Figures 2B–2F). Fire performance was evaluated using a systematic flame spread assessment methodology measuring TTM at 15 mm intervals (Figures 2C and 2D). Detailed experimental procedures are provided in STAR Methods.

### Effects of accelerated weathering on PV backsheet morphology and mechanical properties

Accelerated weathering led to significant “chalking” of PV backsheets, a critical degradation mechanism evidenced by SEM analysis. Chalking refers to the process where surface degradation causes the release of pigment particles, primarily titanium dioxide (TiO_2_),^9^ which has important implications for backsheet durability and potentially its fire resistance. SEM images (Figure 2E) reveal spherical-like structures comparable in size to typical titania pigment particles (0.2–0.3 μm), appearing brighter due to titanium’s higher atomic number compared to the polymer matrix.^9^^,^^10^ The release of these particles gradually erodes the protective layer of the backsheet, potentially exposing underlying layers to environmental stressors and altering the material’s physical structure.

We also observed crack formation in weathered samples (Figure 2E), another crucial degradation feature. These cracks in the external layer not only accelerate deterioration but also compromise insulation effectiveness. More severe cracking can lead to delamination, causing separation of backsheet layers.^11^ The combination of chalking and cracking significantly impacts the backsheet’s protective function, potentially reducing its lifespan, efficiency, and safety.

To quantify the impact of weathering on the mechanical properties of PV backsheets, we conducted tensile testing (Figure 2F) using five samples per condition to ensure statistical validity according to ASTM D638 standards. Our results reveal a clear correlation between weathering exposure and mechanical property degradation. After six weeks of UV exposure, both samples exhibited decreased elongation at break (strain). Sample A’s strain reduced from 126% to 114%, while sample B’s strain decreased from 136% to 124%. These reductions directly reflect the material degradation caused by weathering effects.

The observed changes in mechanical properties can be attributed to polymer chain scission through hydrolysis, a process exacerbated by the moist conditions in our weathering protocol, particularly at temperatures exceeding the polymer’s glass transition point. This hydrolytic degradation leads to a decrease in molecular weight and the formation of various end-groups on the polymer chain.^12^ The process begins with the scission of ester linkages, resulting in reduced molecular weight and an increase in carboxyl end-groups.^13^

Furthermore, tensile strength (Table S1) showed a reduction in both samples, with sample A decreasing from 173.02 to 168.2 MPa (2.8% reduction), and sample B from 112.89 to 92.64 MPa (17.9% reduction). The more pronounced decrease in sample B suggests its KPK structure may be more susceptible to weathering degradation than sample A’s TPE composition. Interestingly, the strain values for both samples showed a slight increase between 4.5 and 6 weeks of weathering. This phenomenon represents “intermediate ductility recovery” in polymer degradation, occurring through three distinct phases: phase I (0–3 weeks) involves primary chain scission that reduces strain capacity; phase II (3–4.5 weeks) shows stress relaxation and increased chain mobility that temporarily improve deformation capacity; and phase III (4.5–6 weeks) where continued degradation overwhelms the temporary recovery. The quantitative evidence supports this mechanism, with aample A strain values of 126% (0 weeks) → 117% (3 weeks) → 125% (4.5 weeks) → 114% (6 weeks), where the 4.5-week value falls within one standard deviation of initial measurements. Similar non-linear degradation patterns have been documented by Sammon et al.^12^ and Zhang et al.^14^ for UV-exposed PET materials, with Uličná et al.^15^ reporting comparable behavior in PV backsheet weathering studies. Importantly, our FTIR spectroscopy analysis shows continuous chemical degradation throughout all time points, confirming this mechanical behavior occurs within an overall degradation trend.

Our findings suggest that during the weathering process, the mechanical durability of the samples is largely retained until the molecular weight is reduced to the transient-molecular-weight region. This provides valuable insight into the degradation timeline of PV backsheets under environmental exposure.

The observed strain increase between 4.5 and 6 weeks represents well-documented competing degradation mechanisms in polymer weathering. During early weathering stages (0–3 weeks), cross-linking reactions dominate, causing material embrittlement and strain reduction. At intermediate stages (3–4.5 weeks), chain scission processes become more prominent, temporarily offsetting cross-linking effects and causing slight strain recovery. This phenomenon, termed “intermediate ductility recovery,” has been extensively documented in PET degradation studies by Gok et al.^16^ and Maddison et al.^17^ Studies by Zaghdoudi et al.^18^ found that “chain scission and cross-linking are occurring with approximately equal impact at shorter aging times,” while for longer aging times, cross-linking dominates, while the influence of chain scission reactions competes with cross-linking.

The competing mechanisms operate as follows: UV radiation initially promotes cross-linking between polymer chains, reducing molecular mobility and decreasing strain capacity. Simultaneously, photolysis and hydrolysis cause chain scission, reducing molecular weight and increasing chain mobility. The relative dominance of these mechanisms shifts with exposure time, creating non-monotonic mechanical property evolution. Additionally, humidity in our weathering protocol enhances chain scission through hydrolytic degradation, particularly affecting the vinyl-ester bonds at elevated temperatures above the polymer’s glass transition point.^19^ UV exposure further creates an overall increase in chain rigidity while causing shortening of polymer chains due to intra-chain scissions.^20^

### Chemical degradation of PV backsheets under accelerated UV weathering

**Figure 3:**
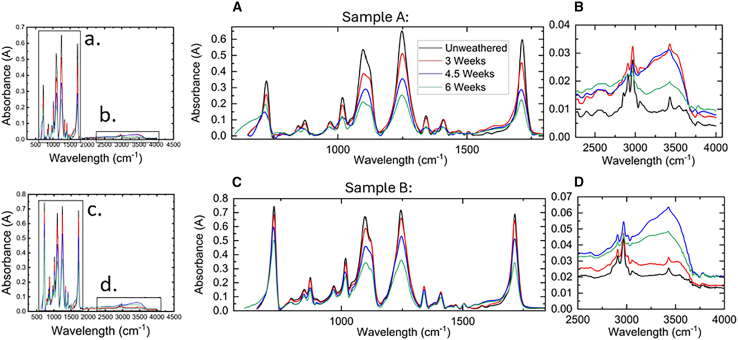
FTIR spectral analysis of chemical changes in weathered PV backsheets (A) Sample A spectra from 500 to 2,000 cm^−1^. (B) Sample A spectra from 2000 to 4,000 cm^−1^. (C) Sample B spectra from 500 to 2,000 cm^−1^. (D) Sample B spectra from 2,000 to 4,000 cm^−1^. Sample A is TPE PV backsheet and sample B is KPK PV backsheet.

Chemical degradation of both PV backsheet samples following exposure to UV radiation and condensation cycles was analyzed using attenuated total reflectance FTIR (ATR-FTIR) spectroscopy (Figure 3). This analysis focused exclusively on the air side, as it is the only part exposed to UV irradiation and condensation. Samples were examined at specific intervals: unweathered, 3 weeks, 4.5 weeks, and 6 weeks.

Our analysis revealed notable spectral changes in both samples, indicating significant chemical degradation that correlates with the observed chalking and mechanical property changes. Key findings include the following.

1. Changes in -OH stretching vibrations: Spectral alterations between 3,510 cm^−1^ and 3,700 cm^−1^^14^ (Figures 3B and 3D) indicate modifications in hydroxyl groups, likely due to hydrolysis of the PET polymer.
2. Formation of chemical groups: The appearance of a peak at 2,928 cm^−1^ suggests the formation of Ar-CH_3_ groups, resulting from PET photolysis.^21^ This indicates chain scission and restructuring of the polymer backbone.
3. Depopulation of -OH bonds: The disappearance of the peak at 3,490 cm^−1^^22^ after weathering signifies a reduction in -OH bonds in PET, consistent with the degradation processes leading to chalking.
4. Progressive degradation: Sample B showed significant changes at 3,430 cm^−1^^14^ after 6 weeks of exposure, with minimal changes at 3 weeks, demonstrating that degradation intensifies over time.

Both samples exhibited considerable chemical degradation following 4.5 weeks of UV exposure and condensation cycles, with peak intensities decreasing as weathering time increased (Figures 3A and 3C). This degradation aligns with the PET air side layer composition confirmed through comparative analysis with existing literature.

These chemical changes provide molecular-level evidence for the mechanisms underlying the observed chalking and mechanical property degradation. The hydrolysis and photolysis of PET, indicated by the FTIR spectroscopy results, directly contribute to the breakdown of the polymer structure, leading to the release of particles (chalking) and the reduction in tensile properties observed earlier.

### Correlation between accelerated weathering and flame spread dynamics in PV backsheets

**Figure 4:**
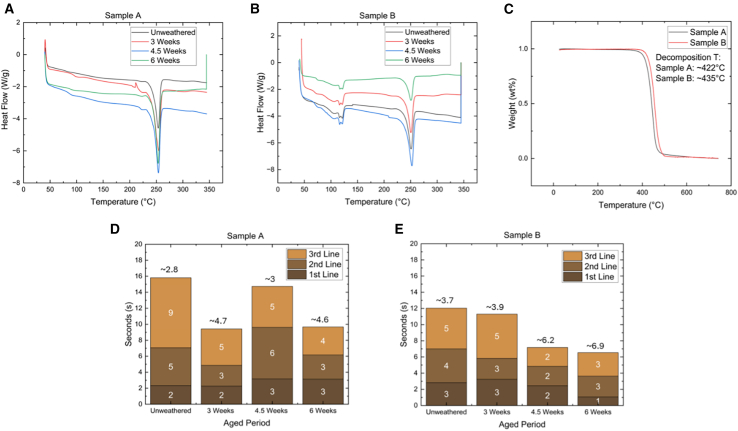
Thermal characteristics and fire performance (A and B) DSC thermograms of samples A and B after weathering. (C) TGA curves of unweathered samples. (D and E) Flame test results showing flame speed (mm/s) as a function of weathering time for samples A and B. Sample A is TPE PV backsheet and sample B is KPK PV backsheet.

DSC was conducted on both samples (Figures 4A and 4B) to assess changes in thermal properties after weathering.^15^ Sample A showed a slight reduction in melting point from 253.59°C to 253.43°C after six weeks of exposure, indicating minor alterations in thermal stability. In contrast, sample B, with its multi-layered composition, exhibited two distinct melting points at approximately 120°C and 250°C. Sample B also showed an increase in melting enthalpy from 28.27 to 28.57 J/g, potentially due to UV-induced crystallization^14^ that may enhance its thermal energy absorption capacity.

TGA revealed the onset of decomposition for sample A at 422.48°C and sample B at 434.92°C (Figure 4C). Weathered samples showed consistent decomposition temperatures with their unweathered counterparts, suggesting minimal changes in thermal decomposition behavior. There are, however, notable differences between the samples, with significant fluctuations evident in the derivative weight curves.^23^

Flame tests revealed significant differences between weathered and unweathered samples. Unweathered sample A exhibited a flame spread rate of 2.8 mm/s (Table S2), taking 9 s to reach the 3rd marker (Figure 4D), the longest time recorded. In contrast, the 6-week weathered sample A showed a rapid overall flame spread time of 10 s, compared to 16 s for the unweathered sample. This indicates that weathering reduces flame propagation time in sample A backsheets.

The relationship between the observed tensile strength degradation and increased flame spread rates can be explained by multiple factors. As tensile strength decreases due to polymer chain scission, the material’s overall integrity is compromised, potentially creating more surface area for combustion and reducing the energy required for degradation during burning. Additionally, the chemical changes identified by FTIR spectroscopy suggest the formation of Ar-CH_3_ end groups that may have different combustion characteristics than the original polymer structure.

Our results demonstrate that weathering significantly alters the fire performance of PV backsheets, generally leading to faster flame spread and easier ignition. This change in fire behavior, coupled with the observed chemical and thermal property changes, underscores the importance of considering weathering effects in the long-term safety assessment of PV installations.

### Implications of weathering on PV backsheet fire performance

Our findings reveal significant changes in the fire performance of PV backsheets after weathering, with implications for long-term PV system safety. The observed increase in flame spread rates, particularly the 46% reduction in TTM for sample B after six weeks of weathering, underscores the critical impact of environmental exposure on fire safety.

The mechanisms behind these changes appear to be multifaceted. FTIR spectroscopy analysis revealed chemical degradation of the PET polymer, including hydrolysis and photolysis, leading to chain scission and the formation of Ar-CH_3_ chemical groups. These chemical changes likely contribute to the observed physical degradation, including chalking and cracking, as evidenced by SEM imaging.

The correlation between chemical degradation and altered fire performance aligns with previous studies on polymer degradation. Our work extends these findings by directly linking weathering-induced changes to fire spread dynamics in PV backsheets, an aspect not previously explored in depth.

Interestingly, while both samples showed chemical and mechanical degradation, sample B exhibited more pronounced changes in fire performance. This difference might be attributed to its multi-layered composition, as revealed by DSC analysis. The presence of two distinct melting points in sample B suggests a more complex degradation process, potentially leading to more significant alterations in fire behavior.

Our results also highlight the limitations of current fire safety standards for PV modules, which typically do not account for long-term environmental exposure. The significant changes observed after just six weeks of accelerated weathering suggest that current testing protocols may not adequately predict the long-term fire performance of PV installations in real-world conditions.

## Discussion

PV systems represent a critical component of global renewable energy infrastructure, but our research reveals a hidden vulnerability that could compromise their long-term safety. By systematically investigating the fire performance of PV backsheets under environmental stress, we have uncovered a concerning transformation of material properties that challenges existing safety assumptions.

Our comprehensive analysis exposes a complex degradation process that fundamentally alters the fire resistance of PV backsheets. Through meticulous examination using FTIR spectroscopy, we observed molecular-level changes that progressively weaken the material’s structural integrity. The current International Electrotechnical Commission (IEC) 61215 standard for PV module durability testing fails to capture these critical long-term degradation mechanisms, potentially leaving critical safety risks unaddressed.

The most alarming finding is the dramatic reduction in flame propagation resistance. After just six weeks of simulated environmental exposure, the backsheet samples demonstrated a 46% increase in flame spread rate, a statistic that should prompt immediate reconsideration of current safety protocols. Tensile strength decreased by up to 18%, while surface morphology changes revealed through scanning electron microscopy highlighted the material’s accelerating breakdown.

These findings extend far beyond academic curiosity. As global renewable energy infrastructure continues to expand, understanding the subtle yet significant material transformations becomes crucial to ensuring public safety. Our research suggests an urgent need to revise existing safety standards, particularly the IEC 61215 standard, to incorporate more rigorous long-term environmental exposure testing.

We propose a comprehensive approach to addressing these critical safety concerns: developing more sophisticated accelerated weathering protocols, implementing mandatory extended-duration testing, and exploring innovative backsheet materials with enhanced fire resistance. The future of renewable energy depends not just on technological innovation, but on our ability to anticipate and mitigate potential risks through meticulous scientific investigation.

This research establishes scientific foundations for improving PV fire safety standards. The demonstrated weathering effects on fire performance highlight critical gaps in current standards that primarily evaluate pristine materials rather than aged components. Our TTM methodology provides a validated framework for component-level fire performance assessment that could be incorporated into future revisions of IEC 61215 or development of PV-specific fire testing standards. The systematic validation approach follows established precedents from fire science literature and provides the scientific basis necessary for potential standardization efforts.

### Limitations of the study

This study provides valuable insights into the effects of weathering on PV backsheet fire performance; however, several limitations should be acknowledged. Our focus on two industrial PV backsheet types, while informative, represents a small subset of commercially available materials. Further research should expand to include a wider variety of backsheet compositions, including fluoropolymer-free alternatives that are gaining market share.

Regarding standardization, we acknowledge that TTM represents a novel testing approach rather than an established standard. However, this reflects critical gaps in current standards rather than methodological limitations. As demonstrated by the successful validation of approaches such as H-TRIS methodology,^24^ scientific validity can be established through systematic validation processes. Our work provides the foundational data necessary for potential standardization efforts, following established precedents where research methodologies eventually become incorporated into standards through demonstrated utility and scientific rigor. The reproducibility concern is addressed through our systematic experimental design including: three replicate samples per condition, standardized sample preparation protocols, controlled flame application procedures, and consistent measurement criteria. Statistical analysis of our results (Table S2) demonstrates acceptable reproducibility with coefficient of variation values comparable to established fire testing methods.

The accelerated weathering protocol, while based on established standards, has inherent limitations in reproducing the complex interplay of environmental factors experienced in field installations. As noted by Uličná et al.,^15^ laboratory accelerated testing may not fully capture regional climate variations that affect degradation patterns. Priority future work includes correlation studies between TTM measurements and full-scale module fire tests to establish conversion factors for field applications. This validation will strengthen the connection between component-level TTM assessment and system-level fire performance in operational PV installations.

Our experimental flame test methodology using conical sample geometry was developed to address the shrinkage issue of thin films during testing. However, as noted by Chen et al.,^25^ sample geometry can affect flame spread dynamics. The conical configuration differs from the flat installation of backsheets in actual PV modules, potentially influencing flame propagation behavior. This geometrical difference should be considered when extrapolating our results to real-world fire scenarios.

The selected weathering duration of six weeks, while sufficient to observe significant changes, represents only an initial period of the potential 25+ year service life of PV modules. Recent research by Zhang et al.^14^ demonstrated that polymer degradation patterns can change over longer exposure periods, suggesting that extended weathering studies would provide more comprehensive insights into long-term fire performance.

For mechanical testing, while our sample size was adequate, increasing the number of replicates in future studies would enhance statistical confidence. Additionally, our focus primarily on UV exposure and condensation cycles excluded other environmental stressors such as thermal cycling, mechanical loading, and pollutant exposure that Kumar and Bhargava^23^ identified as relevant to backsheet degradation.

To address these limitations and advance understanding of PV backsheet degradation and fire performance, we propose several directions for future research.

• Development of more representative flame test methodologies that better simulate the configuration and conditions of installed PV modules, potentially incorporating the influence of mounting systems and air gaps identified as critical by Kristensen et al.^5^
• Investigation of the synergistic effects of multiple environmental stressors on backsheet degradation and fire performance, building on multifactor studies such as those conducted by Kim et al.^13^
• Comparative analysis of weathering effects on other alternative backsheet materials, especially non-fluorinated compositions that may present different degradation and fire performance profiles.
• Examination of weathering effects at module-level fire testing, as component-level testing may not fully capture system-level fire dynamics highlighted by Liciotti.^4^
• Development of predictive models correlating accelerated weathering results with long-term field performance, potentially enabling more accurate lifetime fire safety assessments for PV installations.

These future research directions aim to bridge current knowledge gaps and contribute to enhanced safety standards that account for the effects of environmental exposure on PV system fire performance. As global PV installations continue to accelerate, integrating weathering considerations into safety assessments becomes increasingly important for ensuring the long-term reliability and safety of this critical renewable energy technology.

## Resource availability

### Lead contact

Further information and requests for resources should be directed to and will be fulfilled by the lead contact, Leonard Ng Wei Tat (leonard.ngwt@ntu.edu.sg).

### Materials availability

PV backsheet samples are available from Solar Energy Research Institute of Singapore (SERIS) upon reasonable request to the lead contact.

### Data and code availability

• All data reported in this paper will be shared by the lead contact upon request.
• Any additional information required to reanalyze the data reported in this paper is available from the lead contact upon request.

## Acknowledgments

L.N.W.T. acknowledges Funding from the Singapore Ministry of Education’s Tier 1 grant (RS14/23). We thank Dr. Leow Shin Woei and Dr. Carlos Enrico Cobar Clement for provision of the backsheet samples and for academic discussions.

## Author contributions

The authors of this study are M.M.M.B.A., A.Y.X., R.T.J.H., T.L.S., X.X., M.D., C.Z., and L.N.W.T. M.M.M.B.A. conceptualized the study, developed the methodology, and conducted the investigation. A.Y.X. performed formal analysis and created visualizations. R.T.J.H. contributed to conceptualization, methodology, and investigation. T.L.S. created visualizations. X.X. provided supervision. M.D. handled project administration and supervision. C.Z. contributed to discussions and weathering simulation. L.N.W.T. conceptualized the study, managed project administration, acquired funding, reviewed and edited the writing, and provided overall supervision. All authors contributed to manuscript preparation.

## Declaration of interests

The authors declare no competing interests.

## Declaration of generative AI and AI-assisted technologies in the writing process

During the preparation of this work, the author(s) used ChatGPT in order to proofread and organize content. After using this tool/service, the author(s) reviewed and edited the content as needed and take(s) full responsibility for the content of the publication.

## STAR★Methods

### Key resources table

**Table:** 

REAGENT or RESOURCE	SOURCE	IDENTIFIER
**Experimental models**		
PV Backsheet Sample A (TPE)	Dai Nippon Printing Company	TPE-type
PV Backsheet Sample B (KPK)	Coveme	KPK-type
Atlas UV Test Fluorescent UV Condensation Weathering Device	Atlas Material Testing Technology	UV Test Device
PerkinElmer Frontier ATR-FTIR	PerkinElmer	Frontier
TA Instruments DSCQ10	TA Instruments	DSCQ10
TA Instruments TGA Q500	TA Instruments	TGA Q500
MTS Mechanical Tester	MTS Systems Corporation	Model C42

### Method details

#### Backsheet sample preparation

The samples were obtained from Solar Energy Research Institute of Singapore (SERIS). Sample A is a TPE-type (Tedlar/PET/EVA) backsheet from Dai Nippon Printing Company, while Sample B is a KPK-type (Kynar/PET/Kynar) backsheet from Coveme. These specific types were selected because they represent a significant market share of installed PV modules globally and offer different material compositions for comparative analysis.

#### Accelerated weathering protocol

Accelerated weathering was conducted using an Atlas UV Test Fluorescent UV Condensation Weathering Device, following modified protocols based on IEC 61215 standard test conditions for PV modules. This standard was selected as it is widely recognized for evaluation of polymeric materials in outdoor applications and specifically addresses PV module durability. The samples were trimmed to approximate dimensions to fit into the aluminum specimen holders of the equipment, and sample areas exposed to UV light and condensation measured 95 mm by 63 mm.

The parameters used for accelerated conditions included cyclic exposure to UVA-340A lamps at 1.00 W/m^2^ at 65°C for 8 h, followed by condensing humidity at 50°C in the dark for 4 h, with a water spray duration of 0.15 h. These specific conditions were chosen to simulate multiple weathering stressors (UV radiation, elevated temperature, humidity) that PV modules typically experience in field installations, while accelerating the aging process. The UVA-340A lamps were selected because they provide an excellent simulation of sunlight in the critical short wavelength region from 365 nm down to the solar cutoff of 295 nm, which is the range most responsible for polymer degradation outdoors. The samples were periodically removed from the equipment at intervals of 3, 4.5, and 6 weeks for characterization and flame testing, with these intervals selected based on preliminary testing that indicated observable material changes at these time points.

#### Flame spread assessment methodology

To assess flame spread, we developed an experimental setup comprising two key components: backsheet preparation and testing apparatus configuration. The samples, measuring 63 mm by 95 mm, were subjected to weathering conditions and tested at four intervals: unweathered, 3 weeks, 4.5 weeks, and 6 weeks of exposure.

Sample preparation involved marking three points at 15 mm intervals to quantify flame propagation. To counteract the shrinking effects of the thin polymer sheet under direct flame exposure, we rolled the samples into a conical shape with a 1 mm overlap, secured using metal staples. The conical geometry is necessary because thin polymer films (0.2–0.5 mm) undergo rapid shrinkage and deformation in flat configuration, preventing reproducible measurements. Despite the geometric difference from field installations, TTM measures intrinsic material properties independent of sample geometry.

In the flame test, a controlled flame of 5.5 cm height was applied directly to the bottom center edge of the sample and held for 3 s. In the event that a self-sustaining flame was not observed within the specified period, the flame was removed and reapplied for a second time, held until a self-sustaining flame was observed. We recorded the TTM as the flame reached each 15 mm interval. This methodology was adapted from standard flame propagation test principles in UL 94 VTM while being modified to address the specific characteristics of thin PV backsheet materials. Samples A and B exhibited distinct burning characteristics, and tests were conducted on both unweathered and weathered specimens to ensure consistency and capture any changes in fire performance due to environmental exposure. For each test condition (unweathered, 3 weeks, 4.5 weeks, and 6 weeks), three replicate samples were tested to ensure statistical validity of the results, in accordance with standard practice for fire testing methods.

#### Material characterization techniques

##### Fourier transform infrared spectroscopy

The samples were characterized using a PerkinElmer Frontier ATR-FTIR to observe any changes in chemical degradation. The parameters were set with a wavelength range of 4000 cm^−1^ to 600 cm^−1^, and the number of scans was fixed at 64 with a resolution interval of 4. Both samples were tested with the air side facing down on the diamond sensor hole. The samples were precisely trimmed in order to ensure that only the areas exposed to UV light and condensation were subjected to analysis.

##### Differential scanning calorimetry

To characterize the thermal properties and determine the materials' ability to withstand thermal stresses, the TA Instruments DSCQ10 was employed. The parameters were set to determine the melting points of the samples, with the heating rate set to increase at a rate of 20.00 °C/min up to a temperature of 350.00 °C. Nitrogen was employed as the carrier gas at a flow rate of 50 mL/min. The samples were tested at specified intervals of 3, 4.5, and 6 weeks, in addition to 0 weeks for unweathered samples.

##### Thermogravimetric analysis

The thermal degradation temperatures of the two samples were determined using a TA Instruments TGA Q500. The heating rate for TGA was set to ramp at 20.00 °C/min up to 350.00 °C. The purging and sample gases employed were nitrogen, with flow rates of 40 mL/min and 50 mL/min, respectively. The samples were tested at specified intervals of 3, 4.5, and 6 weeks, in addition to 0 weeks for unweathered samples.

##### Mechanical testing

The mechanical properties of the samples were evaluated using an MTS Mechanical Tester Model C42. The samples were cut into dumbbell shapes using a sheet punching machine in accordance with the ASTM D638 standards for testing plastic materials. Five specimens were tested for each condition to comply with the standard’s minimum sample size requirements. The strain rate of the test was set at 10 mm/min. Tensile strength and elongation at break were measured to quantify the impact of weathering on mechanical integrity, as these properties correlate with material degradation and can influence fire performance through changes in material cohesion and structure.

##### Scanning electron microscopy

Surface morphology analysis was conducted using scanning electron microscopy to observe chalking and crack formation effects on weathered backsheets, providing visual evidence of weathering-induced degradation patterns.

### Quantification and statistical analysis

Statistical analyses were performed using standard analytical methods. Tensile testing used *n* = 5 samples per condition following ASTM D638 standard requirements to comply with minimum sample size specifications. Flame spread tests used *n* = 3 replicate samples per condition to ensure statistical validity of results, in accordance with standard practice for fire testing methods. Data are presented as mean ± standard deviation. For mechanical testing, coefficient of variation was calculated to assess measurement reproducibility. Statistical significance of observed changes was evaluated through comparison of means and standard deviations across weathering time points.

### Additional resources

No additional specialized resources beyond those listed in the key resources table were required for this study.
