# Supplementary material for: Investigating the fire dynamics of mounted PV weathering effects and material changes
Source: iScience. 2025 Aug 29;28(9):113410. doi: 10.1016/j.isci.2025.113410 (PMC12447895; doi:10.1016/j.isci.2025.113410)
Supplement: Document S1. Figures S1–S3 and Tables S1 and S2 [file mmc1.pdf]

iScience, Volume *28*

Supplemental information

Investigating the fire dynamics of mounted

PV weathering effects and material changes

Muhsin Mohamed Baseer Ahamed Mohamed, Ye Xian Ang, Rhonda Jia Hui Tan, Li Song Tung, Xingchi Xiao, Maloy Das, and Leonard Wei Tat Ng

Supplemental Information

Investigating the Fire Dynamics of Mounted PV Weathering Effects and Material Changes

Mohamed Muhsin Mohamed Baseer Ahamed, Ang Ye Xian, Rhonda Tan Jia Hui, Tung Li Song, Xingchi Xiao, Maloy Das, Leonard Ng Wei Tat

Supplemental Figures

Figure S1. Surrounding infrastructure affected due to continuous burning of PV fire, left: Galvanized rooftop deformation, right: Inner steel scaffolding burnt

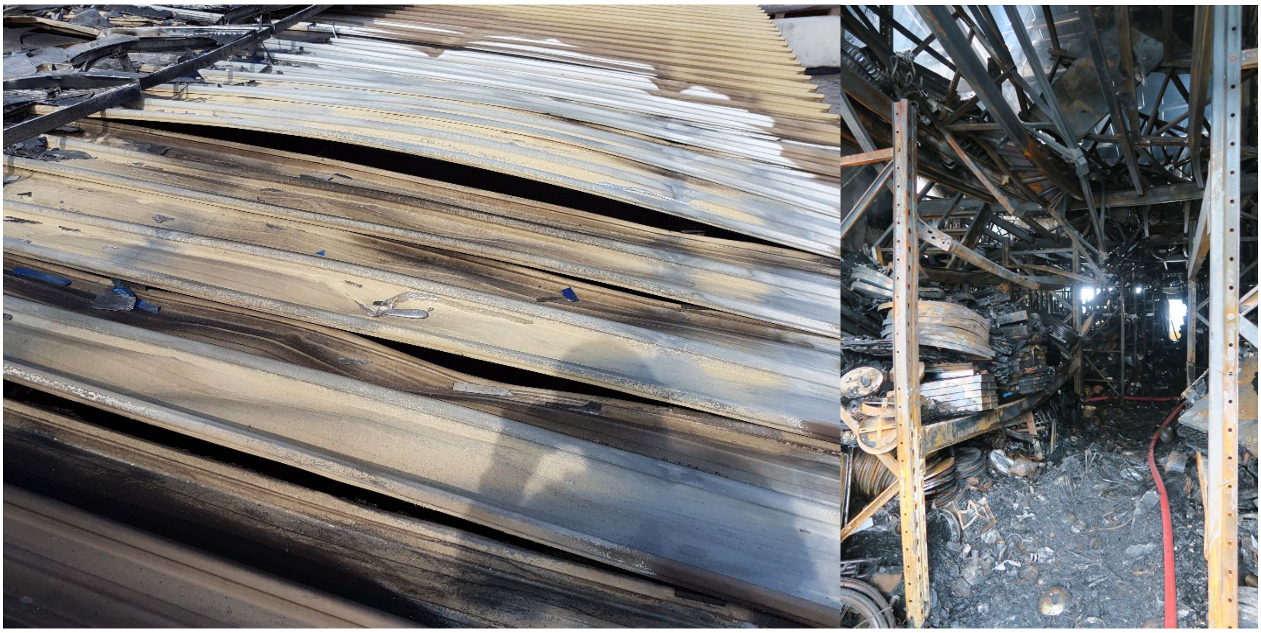

Figure S2. Flame Propagation progresses through the backsheet and the module, leaving behind only the glass components from the front sheet

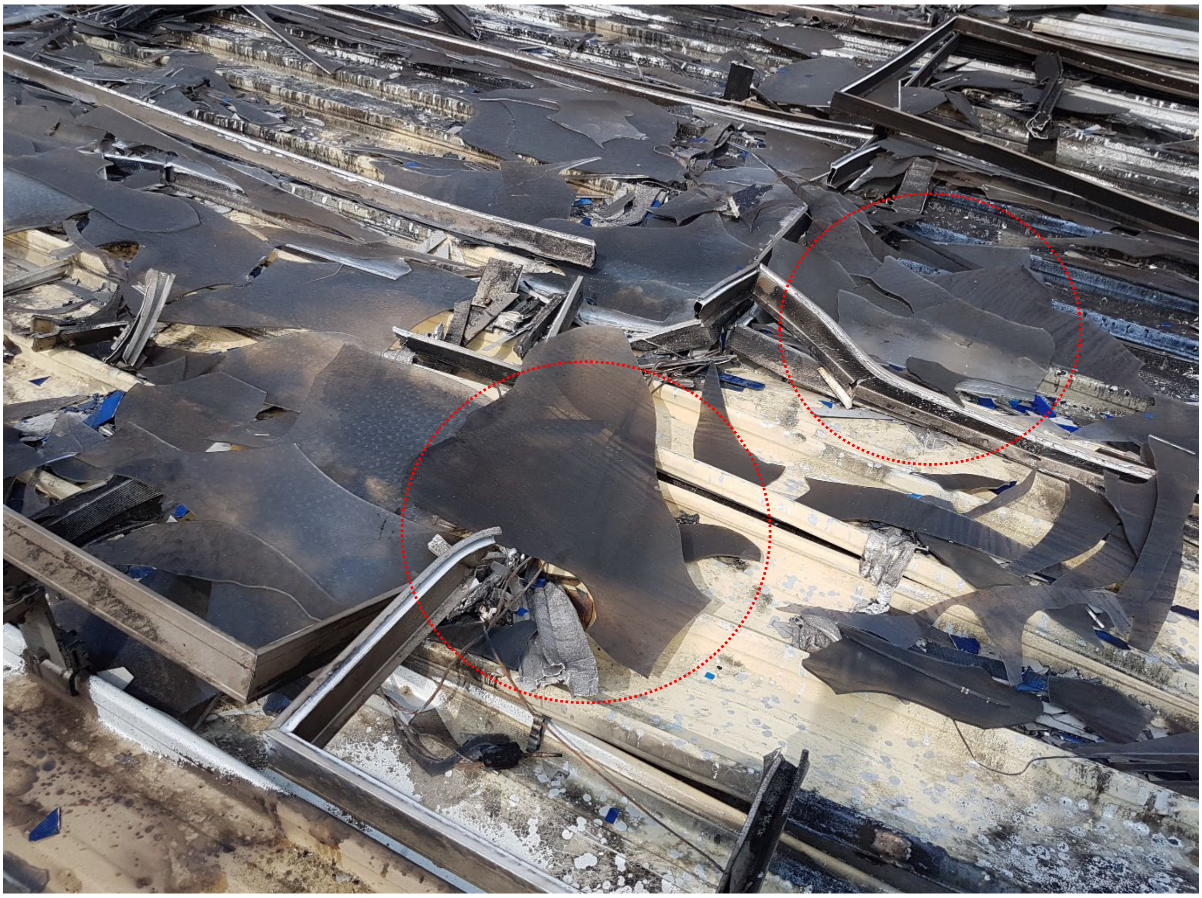

Figure S3. **Crossover of Flame Propagation despite presence of gap**

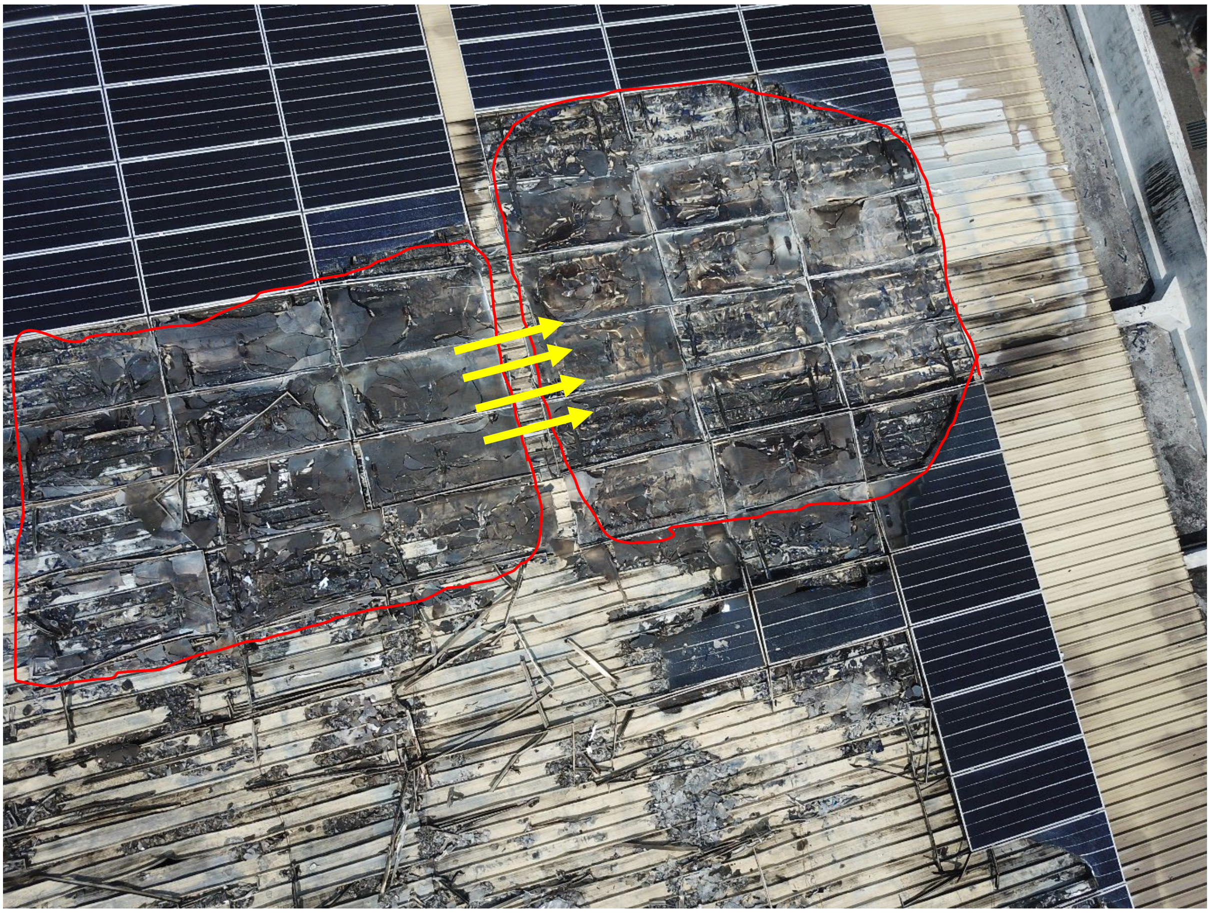

Figure S4. Live Setup & Sample Parameters

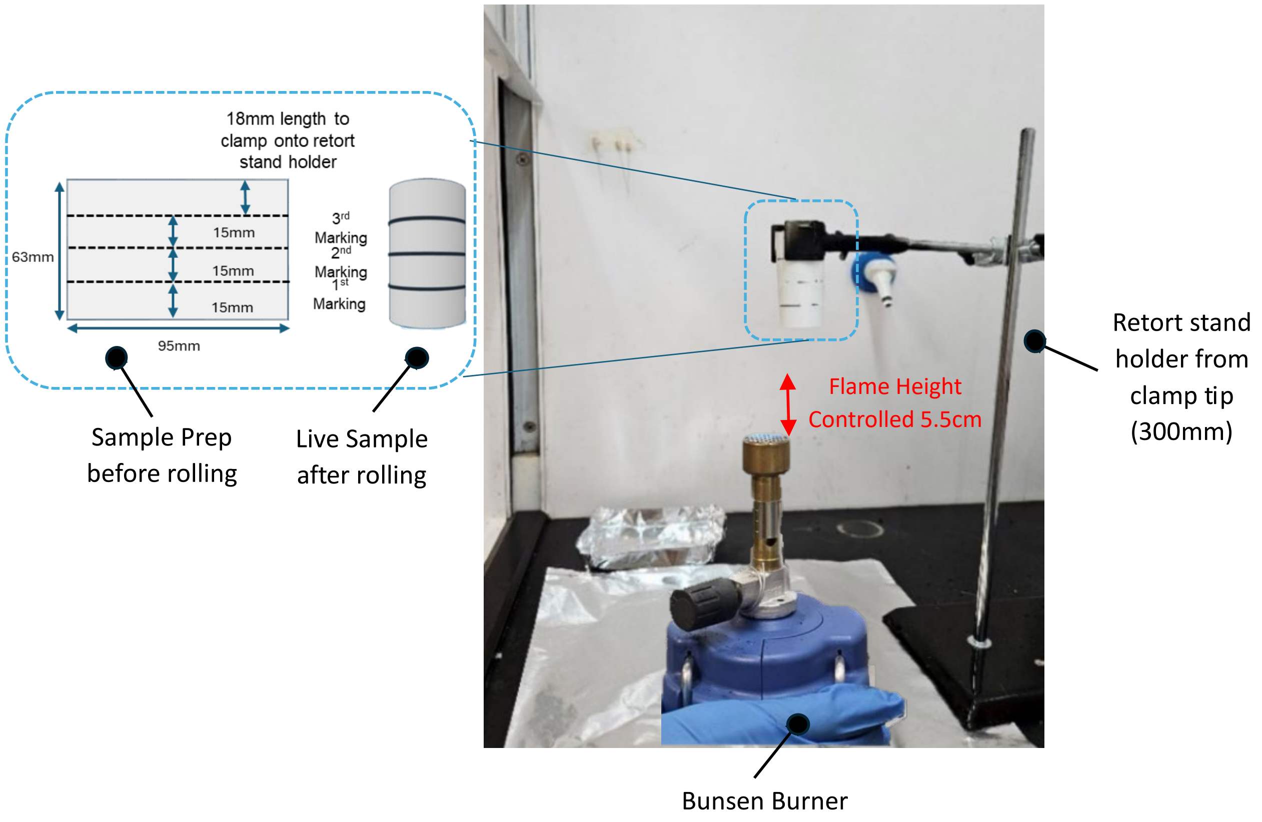

Table S1. **Tensile Strength and EAB values**

	Sample A		Sample B	
**Time**	Strength (MPa)	EAB (%)	Strength (MPa)	EAB (%)
**0 weeks**	173.02	126.22	112.89	136.00
**3 weeks**	166.90	112.33	105.19	127.00
**4.5 weeks**	168.07	114.00	103.70	122.67
**6 weeks**	168.28	114.00	92.64	124.00

Table S2. Calculated flame speeds through sample

	0 weeks	3 weeks	4.5 weeks	6 weeks
Flame Speed (mm/s) across Sample A	2.85	4.79	3.06	4.64
Flame Speed (mm/s) across Sample B	3.75	3.98	6.25	6.92

Table S3. Observations and total taken time after Flame application.

Time	Sample A: Total Flame Propagation Added (seconds)	Sample A: Observations Noted	Sample B: Total Flame Propagation Added (seconds)	Sample B: Observations Noted
0 weeks	16	Ignition observed after the second flame application	12	Ignition observed after the first flame application (within 3 seconds), with minimal dripping
3 weeks	9	Ignition observed after the second flame application, with rapid flame spread	11	Ignition observed after the second flame application, with noticeable increase in dripping
4.5 weeks	15	Ignition observed after the second flame application, with minimal dripping	7	Ignition observed after the second flame application; a longer period required for ignition before sustaining flame
6 weeks	10	Ignition observed after the second flame application	7	Ignition observed after the second flame application

Figure S5. **Experimental limitations of flame test setup**

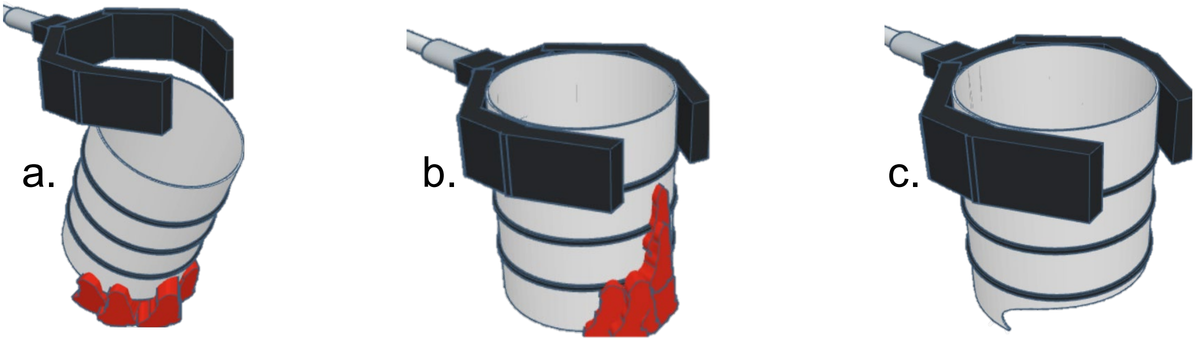

a. **After flame application, sample grip loosens and drops from clamp before reaching 3rd demarcation.**

b. **Propgation of flames found to be too uneven for measurements to be taken, resulting in discrepancies in the timing data obtained.**

c. **After flame application, sample does not ignite and shrinks, less area exposed before 1st marker.**
